# Cardiomiopatia Hipertrófica no Brasil: Avanços no Conhecimento e Implicações Clínicas

**DOI:** 10.36660/abc.20250185

**Published:** 2025-05-29

**Authors:** Danielle Louvet Guazzelli, Mônica Samuel Avila

**Affiliations:** 1 Departamento de Transplante Cardíaco do Instituto do Coração do Hospital das Clínicas da Faculdade de Medicina da Universidade de São Paulo São Paulo SP Brasil Departamento de Transplante Cardíaco do Instituto do Coração do Hospital das Clínicas da Faculdade de Medicina da Universidade de São Paulo, São Paulo, SP – Brasil

**Keywords:** Miocardiopatia Hipertrófica, Prognóstico, Epidemiologia

A cardiomiopatia hipertrófica (CMH) é a doença cardíaca geneticamente determinada mais prevalente e continua sendo um desafio diagnóstico e prognóstico.^1^ A prevalência estimada dessa condição na população em geral é de aproximadamente 1 caso para cada 200 indivíduos.^2^ No Brasil, estima-se que 400.000 pessoas sejam afetadas.^3^

A história natural da CMH é heterogênea, com curso imprevisível que pode se manifestar desde a infância até a oitava década de vida.^4^ A doença também apresenta heterogeneidade fenotípica, com duas formas clínicas principais: obstrução da via de saída do ventrículo esquerdo (OVSVE) e não obstrutiva.

Estudos recentes indicam que aproximadamente 46% dos pacientes podem ter um curso benigno, com expectativa de vida normal. No entanto, outro subconjunto de pacientes pode apresentar sintomas progressivos ou desenvolver complicações clínicas e eventos adversos ao longo do tempo.^5^ Os principais eventos adversos associados à CMH incluem morte súbita cardíaca, isquemia miocárdica, limitação funcional progressiva devido à obstrução da via de saída do ventrículo esquerdo ou disfunção diastólica, progressão para disfunção sistólica do ventrículo esquerdo, fibrilação atrial com risco aumentado de eventos trombóticos e arritmias ventriculares.^6^

A ecocardiografia transtorácica é um método acessível e essencial para o diagnóstico inicial, estratificação de risco e acompanhamento clínico da CMH.^7^ Esse método permite a avaliação da morfologia e função cardíacas, bem como a identificação de fatores prognósticos nessa população.

No Brasil, dados sobre a progressão da CMH em nossa população são escassos, o que reforça a necessidade de pesquisas que reflitam as características específicas dos pacientes no país. Na edição atual deste periódico, os autores do artigo intitulado "Particularidades Clínicas e Ecocardiográficas da Cardiomiopatia Hipertrófica em uma População Brasileira e seu Impacto Prognóstico"^8^ analisaram as características clínicas, ecocardiográficas e a progressão da doença em uma coorte brasileira de pacientes com CMH.

Nesta coorte retrospectiva, 1.244 pacientes com CMH foram analisados durante um período médio de acompanhamento de 7,7 anos. Notavelmente, a hipertrofia septal assimétrica foi o padrão morfológico predominante, observado em 85,4% dos casos, enquanto a OVSVE foi identificada em 30,7% dos pacientes.

O estudo também identificou variáveis associadas a pior prognóstico em pacientes com CMH, incluindo níveis de BNP > 200 pg/mL, diâmetro do átrio esquerdo ≥ 45 mm, fração de ejeção do ventrículo esquerdo ≤ 50%, idade avançada e presença de fibrilação/flutter atrial. A taxa de mortalidade geral foi de 1,3% ao ano, indicando que, embora a CMH seja frequentemente compatível com uma expectativa de vida normal, certos subgrupos de pacientes podem apresentar complicações graves.

Os achados deste estudo brasileiro sugerem diferenças notáveis em comparação com coortes internacionais previamente relatadas. Achados da literatura indicam que a forma obstrutiva da CMH obstrutiva é frequentemente predominante, relatada em até dois terços dos casos.^9^ Em contraste, o presente estudo brasileiro identificou a forma obstrutiva em apenas 30,7% dos pacientes, sugerindo uma menor prevalência da OVSVE em nossa população. Esse achado pode estar relacionado a diferenças genéticas e ambientais ou a limitações metodológicas, como a ausência de testes sistemáticos de manobra de Valsalva para avaliar gradientes dinâmicos.

Em relação ao prognóstico, a taxa de mortalidade anual de 1,3% observada neste estudo é comparável à relatada em coortes internacionais, embora ligeiramente superior. Estudos internacionais relataram taxas de mortalidade anual entre 0,5% e 1,0%^10,11^ ao considerar apenas desfechos cardiovasculares, enquanto o estudo brasileiro analisou a mortalidade por todas as causas, o que pode explicar a discrepância.

Apesar dos valiosos insights fornecidos por este estudo, algumas limitações devem ser consideradas. Por se tratar de um estudo de coorte retrospectivo baseado em prontuários eletrônicos, podem estar presentes vieses de seleção e imprecisões em certas variáveis clínicas e ecocardiográficas. Além disso, a ausência de avaliação sistemática da OVSVE dinâmica e a ausência de métodos complementares de imagem, como a ressonância magnética cardíaca, podem ter subestimado características relevantes, como aneurismas ventriculares e a extensão da fibrose miocárdica.

Apesar dessas limitações, este estudo representa uma das maiores coortes de CMH analisadas no Brasil. Dados epidemiológicos e prognósticos baseados em nossa população são essenciais para orientar a tomada de decisões clínicas e adaptar as diretrizes internacionais à realidade nacional. O reconhecimento de fatores prognósticos específicos em pacientes brasileiros pode contribuir para uma abordagem mais individualizada e precisa do manejo da CMH, melhorando, em última análise, os desfechos e a qualidade de vida dos pacientes.
