# Supplementary material for: Cardiomiopatia Hipertrófica no Brasil: Avanços no Conhecimento e Implicações Clínicas
Source: Arq Bras Cardiol. 2025 May 29;122(4):e20250185. [Article in Portuguese] doi: 10.36660/abc.20250185 (PMC12212368; doi:10.36660/abc.20250185)
Supplement: Translation

# Translation: Hypertrophic Cardiomyopathy in Brazil: Advances in Knowledge and Clinical Implications

**DOI:** 10.36660/abc.20250185i

**Authors:** Danielle Louvet Guazzelli, Mônica Samuel Avila

**Affiliations:** 1 Departamento de Transplante Cardíaco do Instituto do Coração do Hospital das Clínicas da Faculdade de Medicina da Universidade de São Paulo São Paulo SP Brazil Departamento de Transplante Cardíaco do Instituto do Coração do Hospital das Clínicas da Faculdade de Medicina da Universidade de São Paulo, São Paulo, SP – Brazil

**Keywords:** Hypertrophic Cardiomyopathy, Prognosis, Epidemiology

Hypertrophic cardiomyopathy (HCM) is the most prevalent genetically determined heart disease and remains a diagnostic and prognostic challenge.^1^ The estimated prevalence of this condition in the general population is approximately 1 case per 200 individuals.^2^ In Brazil, it is estimated that 400,000 people are affected.^3^

The natural history of HCM is heterogeneous, with an unpredictable course that may manifest from childhood to the eighth decade of life.^4^ The disease also exhibits phenotypic heterogeneity, with two main clinical forms: left ventricular outflow tract obstruction (LVOTO) and non-obstructive.

Recent studies indicate that approximately 46% of patients may have a benign course with a normal life expectancy. However, another subset of patients may experience progressive symptoms or develop clinical complications and adverse events over time.^5^ The main adverse events associated with HCM include cardiac sudden death, myocardial ischemia, progressive functional limitation due to left ventricular outflow tract obstruction or diastolic dysfunction, progression to left ventricular systolic dysfunction, atrial fibrillation with an increased risk of thrombotic events, and ventricular arrhythmias.^6^

Transthoracic echocardiography is an accessible and essential method for the initial diagnosis, risk stratification, and clinical follow-up of HCM.^7^ This method enables the assessment of cardiac morphology and function, as well as the identification of prognostic factors in this population.

In Brazil, data on the progression of HCM in our population are scarce, highlighting the need for research that reflects the specific characteristics of patients in the country. In the current issue of this journal, the authors of the manuscript titled "Clinical and Echocardiographic Particularities of Hypertrophic Cardiomyopathy in a Brazilian Population and its Prognostic Impact"^8^ analyzed the clinical, echocardiographic characteristics and disease progression in a Brazilian cohort of patients with HCM.

In this retrospective cohort, 1,244 patients with HCM were analyzed over a mean follow-up period of 7.7 years. Notably, asymmetric septal hypertrophy was the predominant morphological pattern observed in 85.4% of cases, while LVOTO was identified in 30.7% of patients.

The study also identified variables associated with worse prognosis in patients with HCM, including BNP levels >200 pg/mL, left atrial diameter ≥45 mm, left ventricular ejection fraction ≤50%, advanced age, and the presence of atrial fibrillation/flutter. The overall mortality rate was 1.3% per year, indicating that while HCM is often compatible with a normal life expectancy, certain subgroups of patients may experience severe complications.

The findings from this Brazilian study suggest notable differences compared to previously reported international cohorts. Findings from the literature indicate that obstructive HCM obstructive HCM is often predominant, reported in up to two-thirds of cases.^9^ In contrast, the present Brazilian study identified the obstructive form in only 30.7% of patients, suggesting a lower prevalence of LVOTO in our population. This finding may be related to genetic and environmental differences or methodological limitations, such as the lack of systematic Valsalva maneuver testing to assess dynamic gradients.

Regarding prognosis, the 1.3% annual mortality rate observed in this study is comparable to that reported in international cohorts, though slightly higher. International studies have reported annual mortality rates between 0.5% and 1.0%^10,11^ when considering only cardiovascular outcomes, whereas the Brazilian study analyzed all-cause mortality, which may explain the discrepancy.

Despite the valuable insights provided by this study, some limitations should be considered. As a retrospective cohort study based on electronic medical records, selection bias and inaccuracies in certain clinical and echocardiographic variables may be present. Additionally, the lack of systematic evaluation of dynamic LVOTO and the absence of complementary imaging methods, such as cardiac magnetic resonance, may have underestimated relevant features such as ventricular aneurysms and the extent of myocardial fibrosis.
